# Metabolomic and Genomic Approach to Study Defense Induction by *Nesidiocoris tenuis* against *Tuta absoluta* and *Tetranychus urticae* in Tomato Plants

**DOI:** 10.3390/metabo12090838

**Published:** 2022-09-04

**Authors:** Nomi Sarmah, Athanasios Kaldis, Ioannis Kalampokis, Konstantinos A. Aliferis, Andreas Voloudakis, Dionysios Perdikis

**Affiliations:** 1 Laboratory of Agricultural Zoology and Entomology, Faculty of Crop Science, Agricultural University of Athens, Iera Odos 75, 11855 Athens, Greece; 2 Laboratory of Plant Breeding and Biometry, Faculty of Crop Science, Agricultural University of Athens, Iera Odos 75, 11855 Athens, Greece; 3 Laboratory of Pesticide Science, Faculty of Crop Science, Agricultural University of Athens, Iera Odos 75, 11855 Athens, Greece; 4 Department of Plant Science, McGill University, 21111 Lakeshore Rd., Ste-Anne-de-Bellevue, QC H9X 3V9, Canada

**Keywords:** *α*-linolenic acid, gene expression, jasmonic acid, metabolomics, development, predator

## Abstract

The phytophagy of the predator *Nesidiocoris tenuis* (Hemiptera: Miridae) can trigger defense responses in tomato plants against pests, such as two spotted spider mite *Tetranychus urticae* (Acari: Tetranychidae) and South American leaf miner *Tuta absoluta* (Lepidoptera: Gelechiidae). The expression of genes governing Jasmonic Acid (JA) biosynthesis pathway and fluctuations in the levels of underlying metabolites have been rarely studied in mirid-infested plants. In the present study, fifteen 3rd instar nymphs of *N.* *tenuis* were caged on each top and lower leaf of tomato plants for 4 d to induce plant defense; after this period the predators were removed. With regard to *T. absoluta*, oviposition preference; larval period; and pupal weight were significantly reduced in *N. tenuis*-punctured plants. *T. urticae* adults exhibited a significantly higher escape tendency and reduced survival on punctured plants. Metabolomics confirmed such observations revealing substantial differences between *N. tenuis*-punctured and unpunctured (control) plants. Metabolites directly associated with the activation of the JA defense pathway, such as the precursor *α*-linolenic acid, had increased concentrations. The expression of the defense-related genes *PI-II*, *MYC2*, *VSP2*, and *HEL* was increased in the top leaves and only *VSP2* and *MBP2* in the lower leaves; interestingly, in the middle (unpunctured) leaves *VSP2*, *HEL*, and *MBP2* were also upregulated, indicating systemic signaling. Collectively, phytophagy of *N. tenuis* caused adverse effects on *T. absoluta* and *T. urticae*, whereas the multi-omics approach (phenomics, metabolomics, and genomics) offered valuable insights into the nature of the plant defense responses and provided useful evidence for future applications in integrated pest management, plausibly resulting in the reduction in the required pesticide volumes.

## 1. Introduction

The tomato leaf miner *Tuta absoluta* (Meyrick) (Lepidoptera: Gelechiidae) is the most serious pest of tomato [1,2]. Its control usually relies on intensive insecticide spraying, which could lead to insecticide resistance development [3,4,5]. Due to the shortcomings of chemical control, biological control has been applied, with the zoophytophagous insect predators *Nesidiocoris tenuis* (Reuter) and *Macrolophus pygmaeus* (Rambur) (Hemiptera: Miridae) being frequently employed [6,7]. These predators have several attributes making them effective, such as their polyphagy, plant feeding ability, establishment prior to pest infestation, and efficient prey searching ability [2,8,9,10].

Due to their phytophagous habits, mirid predators wound plant tissues by inserting their mouthparts to ingest plant sap and other nutrients. This wounding process triggers the production of secondary metabolites that are toxic to herbivores and the release of herbivore-induced plant volatiles (HIPVs), which repel pests and/or attract their natural enemies. Tomato plants punctured by *N. tenuis* have been found to be less attractive to whiteflies [11] and *T. absoluta* [12,13], adversely affecting the performance of *Tetranychus urticae* Koch (Acari: Tetranychidae) [14]. Plant feeding by *M. pygmaeus* has negatively affected the reproduction of spider mites and thrips [15,16]. These defensive responses following plant feeding by *N. tenuis* are due to the activation of the abscisic acid (ABA), salicylic acid (SA), and jasmonic acid (JA) metabolic pathways [13]. Previous studies have confirmed that JA and *cis*-12-oxo-phytodienoic acid (OPDA) are accumulated in *N. tenuis* or *M. pygmaeus* infested plants [11,16,17]. Such information has attracted the scientific interest, aiming at developing new approaches in pest control, such as the exposure of plants to predators prior to their field transplantation [13,15,17], which could plausibly result in the reduction in the required pesticide applications.

Phytochemicals, such as secondary metabolites or toxins, provide defense by making the plant tissue less nutritive, and hence less attractive for the herbivore [18]. A plethora of bibliographic references indicates that JA and OPDA play the most prominent role in plant response to herbivory. They belong to the wider family of oxylipins, which are oxygenated compounds, produced in chloroplasts, originating from *α*-linolenic acid [19,20]. 

The production of plant defense metabolites in response to herbivory and the activation of signaling pathways are governed by the expression of defensive genes [21,22]. Proteinase inhibitors (*PI*s), the most studied defense-related proteins in plants [18], are marker genes for JA and have been found to be upregulated in tomato or pepper plants exposed to *N. tenuis* or *M. pygmaeus* [12,14,15,17]. Studies on *Arabidopsis thaliana* and tomato elucidated the mode-of-action (MoA) of JA, of the JA receptor, and of other factors that trigger downstream signal transduction pathways, leading to the induction of defense-related genes [23]. Among them, vegetative storage protein 2 (*VSP2*), myrosinase-binding protein 2 (*MBP2*), hevein-like peptide (*HEL*), and allene oxide synthase (*AOS*) could be explored for resistance. The above-mentioned genes could also be induced by OPDA, although its MoA is not completely understood [19,24,25].

Defense responses may occur in the plant organ originally attacked by a pest (local response) but may also be present in unexposed parts of the same plant (systemic response). Knowledge on the protective systemic effects is essential for future applications in pest control [26]. Systemic response activation by *M. pygmaeus* phytophagy has been reported [15,16]. Zhang et al. [16] concluded that metabolites likely have a major role in the systemic effects. In addition, the increases in JA levels, triggered by phytophagy, may be transient [27], but changes in other metabolites may last for much longer periods [28,29]. Based on the potentially essential role of metabolites in plant defense responses [30], their levels in exposed and non-exposed plant parts have to be investigated. Furthermore, the association between gene expression and metabolite levels, in punctured vs. non-punctured plant parts, will offer the means to develop control measures in pest management, i.e., through classical plant breeding or gene editing technologies [31,32,33,34], or by seed treatment and/or spraying the plants with metabolites [35,36]. However, the changes caused by zoophytophagous predators on the metabolome of exposed plants are still largely fragmented.

Within this context, the aim of the present work was to mine the response of *T. absoluta* and *T. urticae* on tomato plants previously punctured by *N. tenuis*, considering the local and systemic defensive effects, the mapping of the tomato metabolite network, and the transcript analysis of a set of genes involved in the JA defense pathway of tomato within the different plant strata. To our knowledge, this is the first time that the defense induced by the mirid feeding response against the life stages of *T. absoluta* has been investigated in depth. *T. urticae* was also tested as a target pest, with the aim to search the unexplored effects of *N. tenuis*-induced tomato plants on *T. urticae*. Apart from this, it has a different feeding mode than *T. absoluta,* a feature that may affect its response to induce plant defenses [14,16,37]. A multi-omics approach could reveal the fluctuations in the levels of sugars, amino acids, and secondary metabolites, participating in such resistance induction.

## 2. Results

### 2.1. Oviposition Preference of T. absoluta

**Figure 1:**
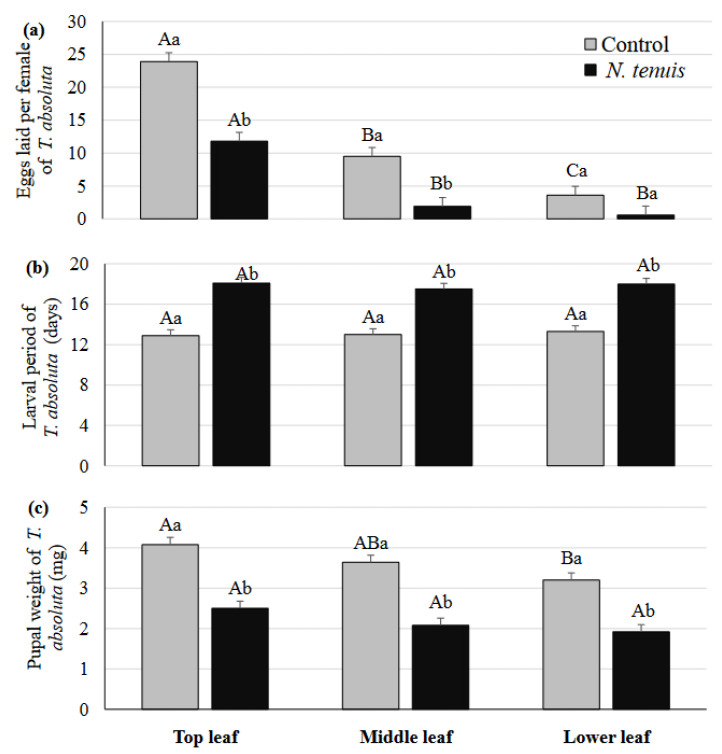
Number (mean ± SE) of eggs oviposited (**a**), larval period, (**b**) and pupal weight (**c**) of *T. absoluta* on top, middle, and lower leaves of tomato plants punctured by *N. tenuis*, in comparison to unpunctured (control) tomato plants. Columns with the same capital letter are not significantly different among leaves within each treatment, and columns followed by the same small letter are not significantly different between treatments within each leaf category (ANOVA, Tukey HSD, *p* < 0.05, *n* = 10).

The oviposition of *T. absoluta* was significantly affected by the “treatment” (*F* = 47.08, *df* = 1,81, *p* < 0.001) and the “leaf position” (*F* = 60.63, *df* = 2,81, *p* < 0.001), with their interaction being significant too (*F* = 5.28, *df* = 2,81, *p* <0.007). The interaction was due to the significantly lower number of eggs laid on the middle and top leaves of the punctured plants, in comparison to the respective leaves of the unpunctured plants (Figure 1a). This indicates a systemic effect, since the middle leaves were not punctured by the predator.

### 2.2. Effects of N. tenuis-Punctured Tomato Plants on Larval Development and Pupal Weight of T. absoluta

No larval mortality of *T. absoluta* was recorded on *N. tenuis*-punctured plants. However, the larval development period was significantly longer on punctured plants (cv. Ace 55) (*F* = 166.34, *df* = 1,27, *p* < 0.001) (Figure 1b). The effects of leaf position and their interaction were not significant (*F* = 0.25, *df* = 2,27, *p* = 0.77 and *F* = 0.21, *df* = 2,27, *p* = 0.82, respectively). The pupal weight was significantly affected by the “treatment” and the “leaf position”, with their interaction not being significant (*F* = 98.76, *df* = 1,27, *p* < 0.001, *F* = 12.85, *df* = 2,27, *p* < 0.001 and *F* = 0.13, *df* = 2,27, *p* = 0.88, respectively). The pupal weight was significantly larger for the larvae developed in the top, middle, and lower leaves of the unpunctured plants than the *N. tenuis*-punctured plants (Figure 1c). The pupal weight was significantly larger in the top than the lower leaves in the control plants.

### 2.3. N. tenuis-Punctured Tomato Plants Induced Escape Tendency of T. urticae

**Figure 2:**
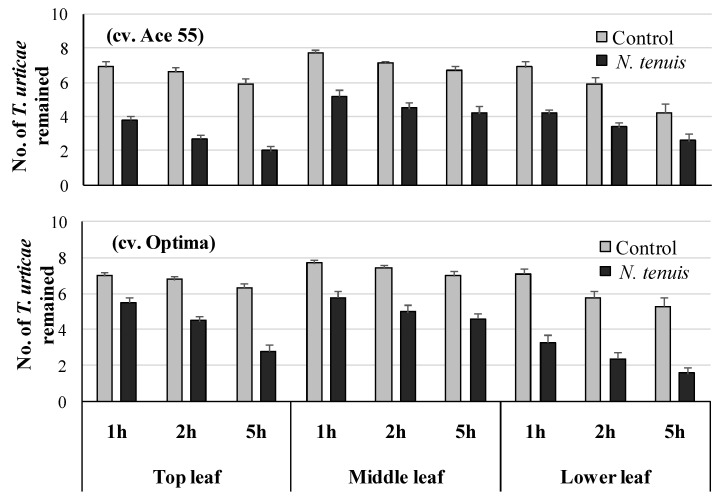
Number of *T. urticae* adults (mean ± SE) remained on leaflets of top, middle, or lower leaves of tomato plants punctured by *N. tenuis* in comparison to unpunctured plants of two tomato cultivars after 1, 2, and 5 h for cv. Ace 55 and cv. Optima. In all cases, a significantly higher number remained on the control than the treated leaf in each time interval in both cultivars. A significantly higher number of adults remained 1 h than at 2 h and 5 h post treatment on the control leaf, in the case of cv. Optima.

The escape tendency of *T. urticae* adults was significantly higher on the *N. tenuis*-punctured than the unpunctured plants in each cultivar (*F* = 29.30, *df* = 1135, *p* < 0.001 and *F* = 33.95, *df* = 1135, *p* < 0.001, for cv Ace and cv Optima, respectively) (Figure 2). It was significantly higher on the top or the lower leaves than the middle leaves (*F* = 11.77, *df* = 2135, *p* < 0.001 and *F* = 12.33, *df* = 2135, *p* < 0.001, for cv Ace and Optima, respectively) and was significantly higher 1 h post treatment (*F* = 36.11, *df* = 2135, *p* < 0.001 and *F* = 39.02, *df* = 2135, *p* < 0.001, for cv. Ace 55 and Optima, respectively). The interaction between the “leaf position” and the “time interval” was significant for cv Ace (*F* = 3.32, *df* = 4135, *p* < 0.01), since the escape tendency was significantly higher on the middle leaf, 5 h after the release of the mites than on the other leaves (Figure 2).

### 2.4. N. tenuis-Punctured Tomato Plants Affect Survival of T. urticae

**Figure 3:**
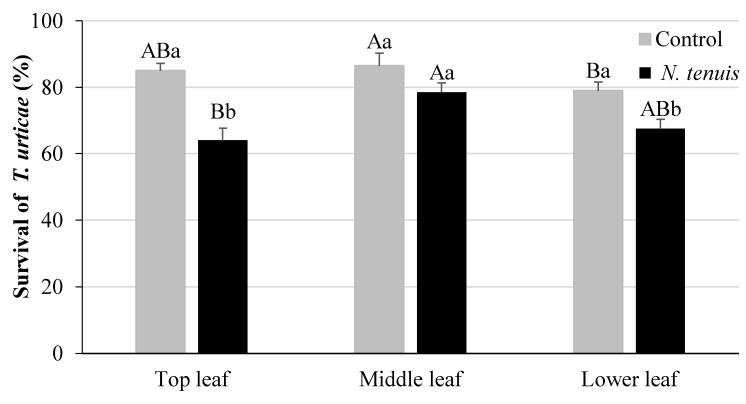
Survival rate (% of alive individuals ± SE) of *T. urticae* 48 h after their release on tomato plants punctured with *N. tenuis*, in comparison to unpunctured plants on top, middle, and lower leaves of tomato plants. Columns with the same capital letter are not significantly different among leaves within each treatment, and columns followed by the same small letter are not significantly different between the treatments in each leaf category (ANOVA, Tukey HSD, *p* < 0.05, *n* = 10).

The survival of *T. urticae* females was significantly affected by the “treatment” and the “leaf position” (*F* = 54.03, *df* = 1,81, *p* < 0.001 and *F* = 7.11, *df* = 2,81, *p* < 0.003, respectively). A significantly higher survival rate was recorded on the top and the lower leaves of unpunctured plants, as compared to the punctured plants (Figure 3). The survival rate was significantly higher in the middle than the lower leaves of the untreated plants; whereas, in the punctured plants, it was significantly higher in the middle than the top leaves.

### 2.5. Overview of the Metabolomics Analyses of N. tenuis-Punctured Tomato Plants

**Figure 4:**
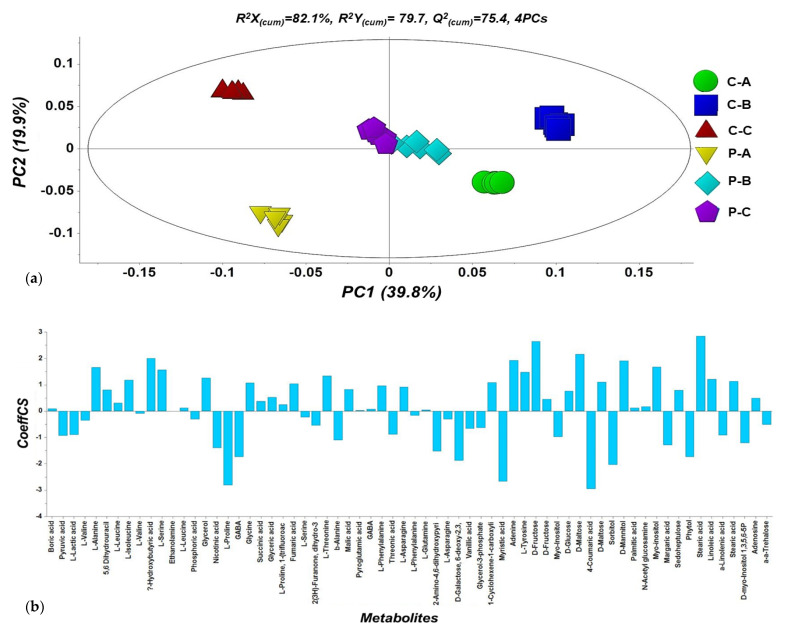
Orthogonal partial least squares-discriminant analysis (OPLS-DA) PC1/PC2 score plot for the GC/EI/MS metabolite profiles of *N. tenuis*-punctured tomato top, middle, and lower leaves, in comparison to the respective leaves of the untreated plants. The ellipse represents the Hotelling’s T^2^ with 95% confidence interval. Six pooled samples were analyzed per treatment (Initial C; control or unpunctured plants, P; plants punctured by predator, A; top, B; middle, C; lower leaves) (**a**,**b**), and OPLS coefficient plot with values of scaled and centered PLS regression coefficients (Coeffs) for the selected Y variables for the whole dataset.

In total, 149 metabolite features were reproducibly recorded, and metabolomics analyses revealed the substantial effect of treatments on 59 metabolites that are related to 43 essential biosynthetic pathways. The differences in the metabolite profiles revealed a strong discrimination among the various treatments (Figure 4a) and the leverage of annotated metabolites are displayed in the corresponding OPLS coefficient plot (Figure 4b).

**Figure 5:**
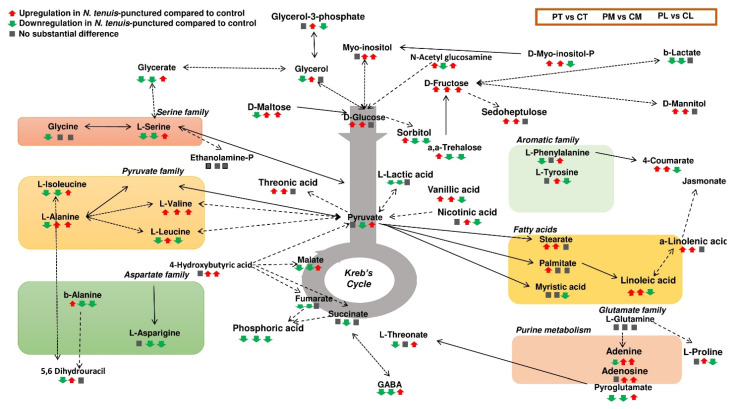
Metabolic network of tomato leaves displaying the differences between the metabolic composition of unpunctured and *N. tenuis*-punctured leaves. Single-headed or double-headed arrows indicate one or two-way reactions between metabolite pools, respectively. Solid lines symbolize one-step consecutive metabolites in a biosynthetic pathway and dashed lines multi-step or not fully elucidated biosynthetic pathway sections. Three different symbols below each metabolite represent their relative abundance in *N. tenuis*-punctured tomato plant tissues (top, middle, and lower leaf), as compared to those in the unpunctured plants. Red upward arrow indicates increased abundance in *N. tenuis*-punctured compared to unpunctured. Green downward arrow indicates decreased abundance in *N. tenuis*-punctured, compared to unpunctured. Gray squares indicate no substantial differences in the two treatments (PT; predator (punctured)-top, CT; control-top, PM; predator-middle, CM; vs control-middle, PL; predator-lower, CL; control-lower leaves).

The *de novo* construction of the metabolic network of *N. tenuis*-punctured tomato plants was based on data retrieved from the Kyoto Encyclopedia of Genes and Genomes (KEGG, https://www.genome.jp/kegg accessed on 19 June 2020) (Figure 5).

**Figure 6:**
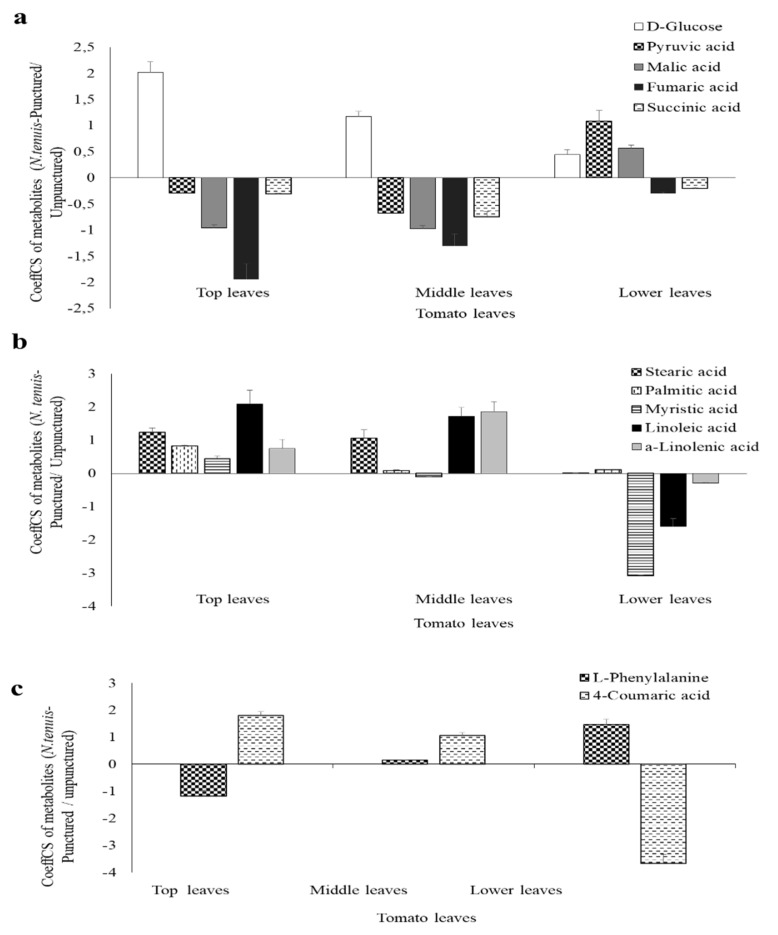
Effect of the *N. tenuis*-treatment in metabolites related to (**a**) glycolysis and Krebs’s cycle (**b**) fatty acids metabolism and (**c**) shikimate pathway. Positive or negative values of scaled and centered regression coefficients (CoeffCS) indicate upregulation or downregulation of the selected metabolites in *N. tenuis*-punctured, in comparison to unpunctured plants, respectively.

A similar fluctuation in the metabolome of top (exposed) and middle (non-exposed) leaves of *N. tenuis*-punctured plants was recorded, in comparison to the lower leaves (Figure 6a). Glucose was detected in high amounts in top and middle leaves of *N. tenuis*-punctured plants. This contrasts with the observed levels of Krebs cycle intermediates, such as pyruvic, malic, fumaric and succinic acids, for which much lower levels were detected in the top and middle leaves of *N. tenuis*-punctured plants (Figure 6a).

Fatty acids such as stearic, myristic, linoleic, and *α*-linolenic acids were recorded in higher levels in the top and middle leaves, compared to the lower leaves of *N. tenuis*-punctured plants (Figure 6b). On the other hand, except for the top leaves, palmitic acid levels did not differ between the middle and lower leaves (Figure 6b). Significantly higher levels of 4-coumaric acid were detected in the top and middle leaves of *N. tenuis*-punctured plants (Figure 6c). Given the fact that L-phenylalanine is the precursor for 4-coumaric acid, it is noteworthy that this metabolite showed downregulation and upregulation in the top and lower leaves of *N. tenuis*-punctured plants, respectively (Figure 6c).

### 2.6. Plant Gene Expression Analysis

**Figure 7:**
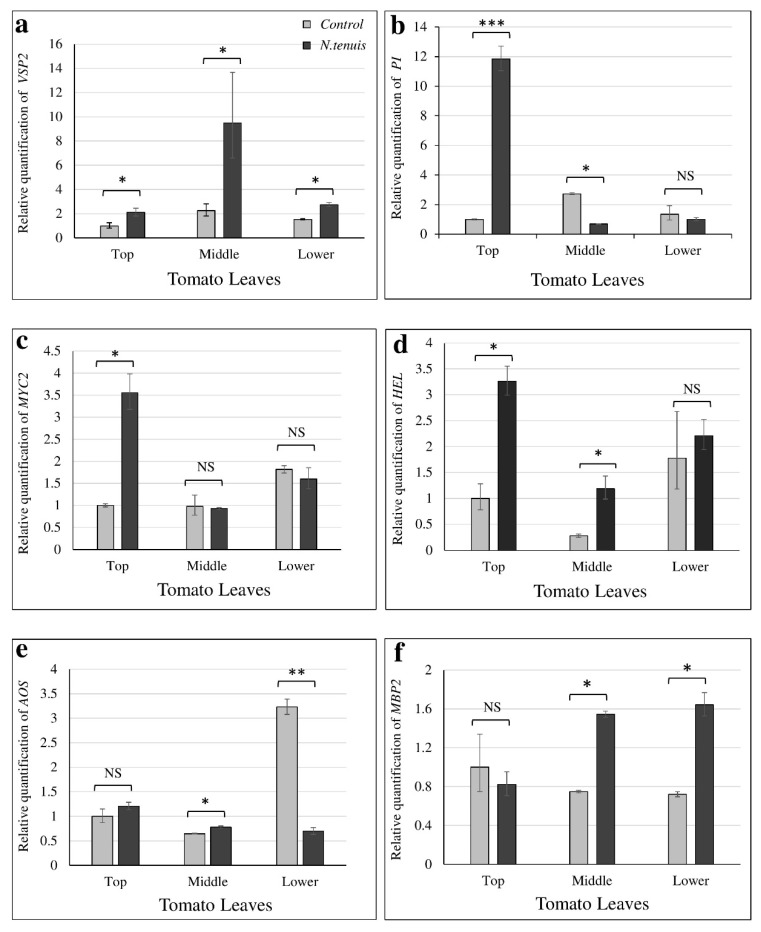
Quantification of defense-related genes affected by the phytophagy of *N. tenuis* on different strata of tomato plants. The relative quantification levels of selected defense-related genes, obtained by RT-quantitative PCR, are shown. Quantification was performed for (**a**) *PI*, (**b**) *VSP2*, (**c**) *MYC2*, (**d**) *HEL*, (**e**) *AOS*, and (**f**) *MBP2*. For normalization purposes, TIP41 was used as the internal control. The 2^−∆∆CT^ method was employed for the quantification. The value obtained for top leaves of unexposed tomato plants was arbitrarily set as 1. Values for all other samples are relative to this. Results were obtained from two biological replicates. Error bars at graphs represent the standard error. Asterisks indicate that the mean expression value in plants exposed to *N. tenuis* is significantly different from unexposed (control) plants (* *p* < 0.05, ** *p* < 0.005, *** *p* < 0.001). NS indicates no statistically significant differences.

The qPCR analysis results of the selected genes indicated a high upregulation (11.84X) for *PI-II* and a significant upregulation for *MYC2* (3.55X) and for *HEL* (3.26X) in the top leaves of *N. tenuis*-punctured, as compared to unpunctured plants (Figure 7). Interestingly, *VSP2* showed a significant upregulation in all leaves (2.1X, 4.22X, and 1.8X in top, middle, and lower leaves, respectively). Significant upregulation in the middle leaves was also observed for *HEL* (4.37X). A significant upregulation was also recorded for *MBP2* in middle (2.1X) and lower (2.27X) leaves of *N. tenuis*-punctured plants (Figure 7).

## 3. Discussion

To our knowledge, this is the first report of the induction of tomato defensive responses against the egg laying and larval development of *T. absoluta* following phytophagy by *N. tenuis*. In a previous study, it was shown that *T. absoluta* adults were less attracted to *N. tenuis*-punctured than unpunctured tomato plants in an olfactometer bioassay [12], most likely due to the emission of repellent volatiles [38]. Similarly, *N. tenuis* treatment reduced the survival rate of *T. urticae*, which agrees with Pérez-Hedo et al. [14] but further induced escape tendency of *T. urticae* adults. Taken together, the results indicate that the tomato defense mechanism triggered by *N. tenuis* has the potential to contribute to the control of *T. absoluta* and *T. urticae.*

A systemic protective response was observed, since the unexposed middle (systemic) leaf of the *N. tenuis*-punctured plants negatively impacted *T. absoluta* at a rate similar to the leaves directly punctured. Such observation is of paramount importance, since the quest for the whole plant protection is desirable. However, regarding the *T. urticae* survival rate, there was less adverse effect on the systemic leaf, as compared to the top (local) leaf. This may be due to the different feeding mode between the two pests (*T*. *absoluta* and *T. urticae*), which might differentially affect the uptake or the differential transport of the deleterious substance for the two pests’ metabolites.

The observed systemic resistance response against *T. aboluta* and *T. urticae* in the middle leaves of *N. tenuis*-punctured plants correlate well with the metabolomics analysis; e.g., the increased levels of metabolites, such as *α*-linolenic and linoleic acids, could be directly associated with the exhibited resistance. Similarly, in the top leaves of *N. tenuis*-punctured plants, increased levels of *α*-linolenic acid were recorded. It has been proposed that the JA pathway is regulated by substrate availability of *α*-linolenic acid [20]. The identification of high amounts of the metabolite in the middle leaves of *N. tenuis*-punctured plants, which were not directly exposed to the predators, indicates the existence of a systemic signal that could trigger the JA pathway in remote plant parts. Hydroperoxide fatty acids, originating from linoleic acid catabolism, may act as long-distance mobile signals that trigger de novo JA biosynthesis in distant parts in cotton [39]. Analyses suggest that the high amounts of linoleic acid in the top and middle leaves of *N. tenuis*-punctured plants might constitute a source of long-distance mobile signals. We propose a model, in which oxylipin intermediates produced in the top leaves get transported to the middle leaves, triggering the JA biosynthetic pathway and also the generation of new mobile signals from linoleic acid catabolism, thus, contributing to the amplification of the defensive response. In line with the proposed model is the observation of relatively high amounts of glycerol-3-phosphate in the middle leaves of the *N. tenuis*-punctured plants, which is an important precursor of numerous metabolites. Chanda et al. [40] presented evidence that glycerol-3-phosphate or a glycerol-3-phosphate-associated factor contributes to systemic immune responses by facilitating the movement of the lipid-transfer protein.

The major effects that the *N. tenuis* phytophagy exerts on plant metabolism are indicated by the increased amount of glucose in the top and middle tomato leaves. Glucose is a substrate for glycolysis, which in association with the Krebs cycle, produces the necessary energy to fuel the growth processes of the plant. The reduced glucose turnover in these tissues is in line with the lower amounts of the intermediary products of the Krebs cycle indicating that the metabolism in the top and middle leaves is redirected from growth and development to defense, as a result of the *N. tenuis* treatment. Sanchez et al. [41] also stated that alanine, leucine, threonine, and glycine had increased concentrations around and below necrotic rings caused by *N. tenuis*, which aligns with our observation. In line with this, metabolomics revealed the activation of the shikimate pathway through phenylalanine, which could be converted to coumarate, i.e., a precursor of anthocyanins, which act as antioxidant compounds in response to various stresses [20]. Taken together, these results suggest a strong link between JA pathway activation and secondary metabolite production is induced in tomato plants punctured by *N. tenuis,* leading to effective local and systemic defense responses against two important tomato pests.

The current study extended our knowledge regarding the array of defense-related genes whose expression is affected by *N. tenuis* punctures in tomato. Previous studies on tomato have focused on the phytohormones JA, ABA, and SA and quantified the transcript levels of PIN2, ASR1 [11], and PI-related genes [14]. With regard to the JA pathway, Pérez-Hedo et al. [11] demonstrated that phytophagy of *N. tenuis* results into high levels of OPDA and isoleucine conjugate of JA (JA-Ile). Zhang et al. [16] reported similar effects when sweet pepper plants were attacked by *M. pygmaeus.* Pérez-Hedo et al. [14] reported upregulated expression of *PIN2* and higher concentration of plant protein inhibitors PI-II1 and PI-II2 via activation of the JA pathway. Similarly, Pappas et al. [15] reported higher accumulation of transcripts of PI genes in tomato plants punctured by *M. pygmaeus.* Since our study showed that *PI-II* was upregulated only in the top leaves, it may finally have a limited contribution to defense against *T. absoluta*. In contrast, the level of upregulation varied among the other genes tested, depending on the plant leaf and if it is punctured or not. In the top leaves (punctured), *MYC2*; *VSP2*; and *HEL* were upregulated and, interestingly, in the middle leaves (unpunctured), *VSP2*; *HEL*; and *MBP2* were, while in the lower leaves (punctured), *VSP2* and *MBP2* were upregulated. The identification of genes that are responsible for the systemic activation of the JA pathway and the protection of the entire plant consist of key knowledge to develop effective pest control methods. The JA- or OPDA-induced gene expression results align well with that of metabolomics data previously discussed.

Our metabolomics results suggested no induction of the JA pathway in the lower (older) leaves, although these leaves were exposed to *N. tenuis*. In agreement, four out of six defense-related genes (*PI-II*, *MYC2*, *HEL*, *AOS*) studied were not upregulated in lower leaves exposed to *N. tenuis*, suggesting that the JA pathway is not induced. In contrast, in younger leaves, which showed the highest photosynthetic capacity, the responses to herbivory were stronger than in older leaves [21,42,43].

Altogether, the results offer valuable information on the selection of the appropriate genes, such as *VSP2, HEL*, and *MBP2,* to offer whole plant protection or long-lasting protective effects in tomato. Future experiments could aid in the production of more resistant plants through gene-editing technologies. CRISPR-dCas9 could be used to activate the transcription of an endogenous gene in a non-transgenic manner [44], e.g., the genes that are upregulated by *N. tenuis* treatment. Delayed development and increased mortality of beetles and the housefly were achieved by incorporating *VSP2* protein into their diets [45]. Additionally, the use of defense-related secondary metabolites, such as stearic, myristic, linoleic, and *α*-linolenic acids, detected at high levels in unpunctured plants when compared to *N. tenuis*-punctured plants and high levels of 4-coumaric acid detected in *N. tenuis*-punctured plants, shown in the present study, may open the potential for novel bio-active compounds to become future plant-derived ecofriendly insecticides [46,47].

Overall, plant feeding by *N. tenuis* can highly influence the performance of *T. absoluta* and *T. urticae* through the activation of the JA pathway, alterations in metabolite levels, and systemic responses. The multi-omics approach (phenomics, metabolomics, and transcriptomics) enabled us to identify metabolites and genes important in plant defense induction. Further studies may follow, investigating the activation of other non JA-mediated pathways potentially involved in pest resistance.

## 4. Materials and Methods

### 4.1. Insect Rearing

*Tuta absoluta* rearing was initiated from adults collected from a tomato crop field located in Marathon, Greece (38°8′24.87″ N 23°58′6.65″ E). The colony was kept in tomato plants cv. Elpida (Spirou House of Agriculture, Athens, Greece) developed from seeds sown individually in plastic seed trays and transplanted after five weeks into 11 cm in diameter plastic pots with compost (Bas Van Burren B.V, The Netherlands). Plants were not sprayed with any pesticide and were kept free from pests and diseases. *T. urticae* rearing was initiated from adults collected from a tomato crop located in Chalkis, Greece (38.46° N, 23.62° E). Bean plants (cv. Barbouni) were used for rearing of *T. urticae.* Rearing of *N. tenuis* (Nesibug, Koppert, Rotterdam, The Netherlands) was kept in tomato plants (cv. Elpida) with “Entofood” (Koppert B.V., Rotterdam, The Netherlands) offered *ad libitum*. Plants and rearings were maintained in entomological cages in an air-conditioned glasshouse at temperature of 25 ± 2.5 °C at the Agricultural University of Athens, Greece.

### 4.2. Exposure of Tomato Plants to N. tenuis

Five-week-old tomato plants of the cv. Ace 55 and cv. Optima (Spirou House of Agriculture, Athens, Greece) with three fully expanded leaves were grown as described above. The use of two instead of a single cultivar was prioritized because we tested the hypothesis mirid bug punctured tomato plants of any cultivar exhibit defense responses with activation of PI genes that have been reported in previous studies (i.e., cv. Optima by Pérez-Hedo et al. [11] and cv. Ace 55 by Pappas et al. [15]); additionally, effects of such defense responses on *T. absoluta* and *T. urticae* were investigated. These results would also be valuable for the wider applicability of the study if there was not an effect of cultivar. Fifteen 3rd instar nymphs of *N. tenuis* were released on each of the top and the lower leaf of each plant. Nymphs were preferred instead of adults because they can induce plant defense [48], and, additionally, such approach prevents oviposition that may also induce defense [49] and emergence of new nymphs. Ten different plants per treatment had been used. Then, each leaf with or without the nymphs was enclosed into an organdy bag (12 × 15 cm). The middle leaf of each plant was enclosed in a bag without any *N. tenuis* nymphs being introduced. No food for *N. tenuis* was added. Each leaf of the control tomato plants was enclosed in a bag individually without *N. tenuis* nymphs. All the experiments were conducted at 25 ± 1 °C, 65 ± 5% RH and 16:8 h photoperiod (light: dark). After four days of exposure, the survival of the predators was always higher than 80%.

### 4.3. Effects on Oviposition, Larval Development Period, and Pupal Weight of T. absoluta due to Exposure to N. tenuis

After the removal of the predators, a punctured tomato plant was introduced in a cage (35 × 35 × 60 cm) (BioQuip, Compton, CA, USA) together with an unpunctured one, both of either the cv. Ace 55 or cv. Optima. Then, three pairs of *T. absoluta* adults (less than 48 h old) were introduced into the cage using a mechanical aspirator. Plants were kept without touching either each other or the cage walls. The adults were allowed to oviposit for the next 24 h. Then, the number of eggs oviposited on each leaf of both plants was recorded. Ten replicates (cages, with one punctured and one control plant) were used per cultivar.

Ready-to-hatch eggs (4d-old) of *T. absoluta* were placed carefully on each top, middle, and lower leaf (one egg per leaf) of *N. tenuis*-punctured and unpunctured plants. The mortality was monitored daily for each larva during its development on each leaf. Upon molting into pupa, the pupal weight was recorded using an analytical balance (KERN ACS 80-4, Stuttgart, Germany). In this case, only one cultivar (cv Ace 55) was used because the effect of cultivar was not found significant on the oviposition rate of *T. absoluta*. Ten punctured and ten control plants were used.

### 4.4. Effects on Escape Tendency and Survival of T. urticae due to Exposure to N. tenuis

Following the removal of *N. tenuis*, 10 (5 female and 5 male) young adults (3–6 days old) of *T. urticae* were placed on a leaflet of each top, middle, and lower leaf of *N. tenuis*-punctured and unpunctured tomato plants of both cultivars using a fine brush. *Tetranychus urticae* escape tendency was recorded by counting the number of adults that remained on the leaflet 1, 2, and 5 h later. The effects on the survival rate of the *T. urticae* adults were assessed using 5 female and 5 male adults of less than 24 h in the adult stage and previously starved for 2 h. The adults were confined within a circular area of 3 cm in diameter on the adaxial surface of a tomato leaflet of each leaf of a tomato plant by the aid of entomological glue (Temo-O-Cid Glue, Verde Vivo Company, Vigonovo, Italy). After 48 h, their survival was recorded based on the dead adults found in the circular area. Ten punctured and ten control plants were used as replicates for each cultivar.

### 4.5. Sampling and Sample Preparation for GC/EI/MS Metabolomics

Fifteen 3rd instar nymphs of *N. tenuis* were released on each top and lower leaf of five-week old tomato plants of cv. Ace 55 for four days. Top, middle, and lower leaves of six biological replicates per punctured and unpunctured plants were harvested and immediately flash frozen in liquid N_2_ for metabolism quenching in 50 mL falcon tubes. The extraction of the tomato leaf metabolome was performed, as previously described, with minor modifications [50]. Pulverized leaf tissues (40 mg) were transferred into 2 mL Eppendorf tubes and the extraction was performed by adding 500 μL of a methanol-ethyl acetate (50:50 *v*:*v*) mixture. The resulting suspensions were sonicated, stirred, and filtered through PTFE filters (0.2 μm diameter pore, Macherey-Nagel, Duren, Germany). Filtered extracts were spiked with 20 μL of a ribitol solution (0.2 mg·mL^−1^ in methanol) (Sigma-Aldrich Ltd., Steinheim, Germany), which was used as an internal standard. The extracts were evaporated to dryness and derivatized by applying a two-step process [50,51] using a solution of methoxylamine hydrochloride in pyridine (98% *w*/*w*) (Sigma-Aldrich Ltd., St. Louis, MO, USA) for methoxymation and N-Trimethylsilyl-N-methyl trifluoroacetamide (MSTFA) for silylation. Blank samples were similarly analyzed to monitor metabolite features not related to the analyzed plant material. Furthermore, analytical standards were analyzed for the absolute identification of selected metabolites (Sigma-Aldrich Ltd.).

### 4.6. GC/EI/MS Metabolite Profiling of Tomato Leaves and Data Pre-Processing

The metabolite profiling of tomato leaves was performed using an Agilent 6890 analytical platform (Agilent Technologies Inc., Santa Clara, CA, USA) (5973 series mass selective detector, 7683 autosampler). The settings of analyses have been previously described [50,51]. Briefly, 1 μL of the samples were injected on a column [HP-5MS, length; 30 m, i.d.; 0.25 mm, film thickness 0.25 μm (Agilent Technologies Inc., USA)] with the injector set to a 10:1 split mode. Helium was used as the carrier gas at a flow rate of 1 mL·min^−1^ and full scan mass spectra were acquired at the mass range of 50–800 Da (scan rate of 4 scans·s^−1^).

The acquired total ion chromatograms were deconvoluted using the software AMDIS v.2.66 (NIST, Gaithersburg, MD, USA) and data pre-processing was performed by the bioinformatics software MSDIAL v.4.38 [52,53]. The aligned data were exported to MS Excel^®®^ for curation and further examined for inconsistencies [50,51]. Metabolite features present in less than 50% among replicates were excluded from further analyses. Additionally, metabolite features also detected in the experimental blank samples were removed during matrix curation and were excluded from further processing.

Tentative metabolite identification was based on matching their mass spectra and retention times to reference entries of the Golm Metabolome Database [54] and the National Institute of Standards and Technology library ’08 (NIST 08, Gaithersburg, MD, USA) (mass spectra similarity > 95%), and for selected metabolites absolute identification was performed using analytical standards. For the biological interpretation of the results and the discovery of trends and the corresponding biomarkers using the software SIMCA-P v.13.0 (Umetrics, Sartorius Stedim Data Analytics AB, Sweden), a previously described approach was adopted [51,55] with minor modifications. Briefly, multivariate analysis was performed, and the discovery of tomato metabolites biomarkers was based on OPLS-DA regression coefficients (Coeffs) (*p* < 0.05). Standard errors were calculated using jack-knifing. Metabolites with values of Coeffs > 1 and Coeffs < −1 were considered as biomarkers.

### 4.7. RNA Extraction and Plant Gene Expression Analysis

In order to investigate the systemic effect caused by *N. tenuis* phytophagy, the upregulation of anti-herbivory genes *PI-II*, *MYC2*, *VSP2*, *HEL, AOS*, and *MBP2* was investigated. Fifteen 3rd instar nymphs of *N. tenuis* were released on each top and lower leaf of five-week-old tomato cv. Ace 55 plants for four days. Six *N. tenuis*-punctured tomato plants and six unpunctured (untreated) plants were employed for gene expression analysis. Upon completion of the 4-day treatment, bulk samples of 200 mg tomato leaf tissue from three plants per treatment (two independent biological replicates) were collected separately from the top, middle, or lower leaves of *N. tenuis*-punctured and unpunctured plants. Leaf tissue was flash frozen in liquid nitrogen to stop gene expression and total RNA isolation was performed using TRIzol by adapting the Yoo et al. [56] procedure. RNA concentration was measured spectrometrically and it was adjusted to 100 ng·μL^−1^ with RNase-free water. An amount of 1–2 µg RNA from each sample was electrophoresed at 135 V for 35 min in 1.5% agarose gel. The integrity of the ribosomal bands confirmed the quality of RNA. Reverse transcription (RT) was performed, employing an oligo-dT primer and FIREScript Reverse Transcriptase, Solis BioDyne, Estonia, following standard protocol [56].

Several genes, known to participate in the JA pathways, were selected for expression analysis (Table 1) in order to investigate the involvement of these genes to *N. tenuis* phytophagy in tomato. Since the pathways are not fully characterized in tomato, the gene sequence information for the selected and well-characterized genes in *Arabidopsis thaliana* [19,24] was retrieved from the TAIR database (https://www.arabidopsis.org/ accessed on 15 November 2018), as *Arabidopsis thaliana* is one of the most thoroughly studied plant species. In fact, the elucidation of the mode of action of jasmonic acid, the identification of the jasmonic acid receptor, and of other protein factors that trigger downstream signal transduction pathways leading to the induction of defense-related genes have all been achieved based on studies on *Arabidopsis thaliana*. This approach may provide a wider pool for selection of genes to edit in potential future applications. Next, these sequences were used to perform BLAST (Basic Local Alignment Search Tool) search in the SGN database (https://solgenomics.net/ accessed on 22 November 2018) and to identify the ortholog genes in tomato. Specific primers for the tomato genes were designed employing the Primer 3 (http://bioinfo.ut.ee/primer3-0.4.0/ accessed on 1 December 2018) (Table 1).

For quantification of the expression levels of the selected genes, quantitative PCR (qPCR) reactions were performed with the help of 5X HOT FIREPol EvaGreen qPCR Supermix (Solis BioDyne, Tartu, Estonia), in a StepOnePlus Real-Time PCR System (Applied Biosystems, Bedford, MA, USA). For the relative quantification, the 2^−∆∆CT^ method was employed [57], using *TIP41* as the housekeeping gene *TIP41* (TAP4 interacting protein of 41 kDa) for normalization purposes [58]).

### 4.8. Data Analysis

The number of eggs laid by *T. absoluta* were analyzed with a mixed model with fixed factors being the “treatment” (i.e., *N. tenuis*-punctured vs. unpunctured plant), the “cultivar” (Ace 55 vs. Optima), and the “leaf position” (i.e., top, middle, and lower). Then, aiming to control for plant and leaf position variation we included in the model “plant” X “leaf position” as nested random effects. Data of its larval developmental period and the pupal weight were similarly analyzed with fixed factors being the “treatment” (i.e., *N. tenuis*-punctured vs. unpunctured plant) and the “leaf position” (i.e., top, middle, and lower). Raw data were log transformed prior to the analysis. The escape tendency of *T. urticae* individuals was estimated as the percentage of individuals escaped from the leaflet at each time interval. The data were arcsine transformed and compared within each tomato cultivar with fixed factors being the “treatment”, the “leaf position”, and the “time interval”, and “plant” X “leaf position” as nested random effects. The data of *T. urticae* adults’ survival 48 h after their release were analyzed following the same methodology with factors being the “cultivar”, the “treatment”, and the “leaf position” after data were arcsine transformed. In all cases, means were compared using the Tukey’s HSD test (*p* < 0.05).

For gene expression analysis, significant differences were determined with a Student’s t-test performed in a pairwise manner by comparing the gene expression levels in plants punctured by *N. tenuis* or unpunctured on the same strata (top, middle, or lower leaves). The results are presented as the mean values from two biological replicates ± standard error (SE). Statistical analyses were performed with JMP 14.0 (SAS Institute Inc., 2016, Cary, NC, USA) [60].

## 5. Conclusions

Plant defense induced by *Nesidiocoris tenuis* in tomato plants caused significant adverse effects on *Tuta absoluta* oviposition and development proving that this method can be valuable in the control of this serious pest. Similarly, *Tetranychus urticae* adults had an increased escape tendency and reduced survival. These effects were proved systemic on two tomato cultivars, further indicating their potential in pest control. Additional research to explore the nature of these plant defense responses in depth showed that metabolites associated with jasmonic acid pathway had increased concentrations on *N. tenuis*-punctured tomato plants. Increased levels of metabolites recorded in the middle (un-punctured) leaves, confirming their role in the observed systemic nature of the responses. The expression levels of several plant-defense associated genes enabled us to identify certain genes responsible for the activation of plant-defenses and metabolite fluctuations on both punctured and un-punctured leaves, thus, having a major role in the plant-defense activation. Overall, the outcomes are complementary to each other, indicating an integrated approach in developing novel strategies in pest control either by activating plant defenses using metabolites as ecofriendly insecticides or through gene editing technologies targeting genes largely involved in plant-defense phenomena. Further studies may follow investigating the development, the practical application, and evaluation of the above approaches in pest control.

## Figures and Tables

**Table 1:** Names of the genes with their respective selection criteria and primers used.

Gene Name		Criteria for Selection	Primers Forward (F) and Reverse (R)	Refs
Proteinase inhibitor II	PI-II	Induced by the JA pathway, harmful for the digestive system of insects	F: GGATATGCCCAGGTTCAGAAGGAA R: AATAGCAACCCTTGTACCCTGTGC	[59]
Vegetative storage protein 2	VSP2	Acid phosphatase and anti-insect activity, specific to JA, induced by MYC2	F: CTGGTTATGCAGTCCCACAAT R: ACGTCGATATTGTTTGCCAAG	This study
Myrosinase-binding protein 2	MBP2	May contribute to the production of toxins protecting against herbivory	F: CACAAACATCAGAGGCCATTT R: TGCACCATGTTTTACTGACCA	This study
Allene oxide synthase	AOS	Component of the JA-biosynthesis pathway, coi1-dependent	F: GATTTCGTTGTGATGGTTTCG R: TCGACGTTGAGTGTACCGTAA	This study
Hevein-like peptide	HEL	Antimicrobial peptide, highly induced by herbivory	F: TGTTGATTATATCCGCGATTG R: TTGGAAGGTGAACAAAATTCG	This study
Myelocytomatosis oncogene transcription factor 2	MYC2	Major transcription factor, component of the JA pathway, anti-insect activity	F: GATGATCCAACAAGCCACAGT R: CGATGTCAACGCTACCCTAAG	This study
TAP4 interacting protein of 41 kDa	TIP41	Housekeeping gene	F: GCTGCGTTTCTGGCTTAGG R: ATGGAGTTTTTGAGTCTTCTGC	[58]

## Data Availability

Data available on request due to restrictions eg privacy or ethical The data presented in this study are available on request from the corresponding author. The data are not publicly available because it is a part of doctoral study of NS which is yet to be presented.
